# Preferences of Sjögren’s syndrome patients regarding potential new saliva substitutes

**DOI:** 10.1007/s00784-022-04576-w

**Published:** 2022-06-11

**Authors:** Zainab Assy, Floris J. Bikker, Esra Mashhour, Mina Asadi, Henk S. Brand

**Affiliations:** Department of Oral Biochemistry, Academic Centre for Dentistry Amsterdam, University of Amsterdam and Vrije University Amsterdam, Amsterdam, the Netherlands

**Keywords:** Sjögren’s syndrome, Dry mouth, Xerostomia, Saliva substitutes, Patient preferences, Ingredients

## Abstract

**Objectives:**

Sjögren’s syndrome (SS) patients should be involved in the development of new saliva substitutes at an early stage. The purpose of the current study was to explore the preferences of these patients regarding various product characteristics of potential new saliva substitutes.

**Material and method:**

A questionnaire was distributed among SS patients. They could anonymously indicate their preferences for saliva substitute characteristics using 5-point Likert scales.

**Results:**

Fifty-nine SS patients filled in the questionnaire. According to their opinion, the most ideal saliva substitute has a thin-watery consistency with a neutral flavour that should be applied as a spray. Patients demand a prolonged alleviation of dry mouth complaints and neutralization of harmful bacteria. The patients mainly object against the presence of artificial sweeteners and alcohol in saliva substitutes, but have limited objections against the presence of vegetable-based ingredients and natural enzymes. Major objections were against the potential side effects “bitter taste” and “discoloration of teeth”. Age and severity of xerostomia affected desire of flavours. Younger patients preferred menthol flavour, while respondents with severe xerostomia preferred the use of “neutral flavours” significantly more.

**Conclusion:**

The most ideal saliva substitute has thin-watery consistency in spray form with a neutral flavour and providing long alleviation of dry mouth complaints. Besides, it should not contain artificial sweeteners or alcohol, and should not have a bitter taste or cause discoloration of the teeth.

**Clinical relevance:**

Investigating the opinion of SS patients provides tailoured insights into their preference, which may contribute to the development of more effective saliva substitutes.

## Introduction

Sjögren’s syndrome is an autoimmune disease that causes a progressive damage to the exocrine glands including the salivary glands. As a consequence, Sjögren’s syndrome leads to hyposalivation and/or xerostomia [1, 2]. The resulting dry mouth may induce comorbidities such as difficulties with mastication, swallowing, speaking, and sleeping. In addition, the reduction of the protective properties of saliva may also increase the risk of developing dental caries and oral candidiasis [1, 3].

At early stages of Sjögren’s syndrome, when residual salivary function is still present, salivary flow can be stimulated, e.g. by the use of lozenges and chewing gums, systemic pharmacotherapy, or electrostimulation of the salivary glands [4–6]. However, in case of an advanced disease process, when the salivary function is irreversibly impaired, saliva substitutes such as mouth sprays, gels, and mouthwashes can be applied for the relief of oral complaints [6, 7]. A substantial number of Sjögren’s syndrome patients is using or has used a saliva substitute in the past. In a recent study, this percentage varied between 42.9 and 45.5% for the use of a mouth gel, while for the use of a mouth spray it varied between 25 and 27.4% [7, 8].

The currently available saliva substitutes contain animal- and vegetable-based lubricants and thickeners like porcine gastric mucins, hydroxyethyl cellulose, or aloe vera [9]. However, these ingredients have limited ability to retain water and/or require specific environmental conditions to be effective. For example, porcine gastric mucins are only effective at an acidic pH and in a low ionic strength environment [9]. Besides, some compounds are easily removed from the oral cavity by swallowing or drinking, leading to limited duration of moistening and lubrication. Additionally, a number of substitutes have flavours such as “apple”, “lemon”, and “strawberry”. A reason for manufacturers to use these flavours is that they potentially can stimulate the salivary secretion due to their gustatory effect [10]. However, more than the half of Sjögren’s syndrome patients reported that they discontinued the use of saliva substitutes after a short period of time. An unpleasant taste and sticky consistency were main reasons for discontinuation of the use of saliva substitutes [11, 12]. The sticky consistency may compromise masticatory function [13]. Also, the presence animal-based products in salivary substitutes could induce objections in people from certain religious, cultural, and social backgrounds because these products may be against their beliefs [14].

However, to the best of our knowledge, no studies have been conducted to investigate patients’ preference for characteristics of saliva substitutes, such as taste, consistency, and objections for specific ingredients. In our view, investigating the opinion of the users at an early stage of the development of new saliva substitutes might provide tailoured insights into preference criteria which may contribute to the development of more effective saliva substitutes. For this reason, the purpose of the current study was to explore the preferences of Sjögren’s syndrome patients regarding various product characteristics of potential new saliva substitutes, especially important functions of possible substitutes, objections against certain ingredients, desired flavours for the substitutes, objections against potential side effects of saliva substitutes, and the preferred method of administration. The unpleasant taste of saliva substitutes is a major reason for discontinuation of use of these products [11, 12]. Therefore, we explored the preferences of a wide range of possible flavours. As the amount of saliva present in the oral cavity may affect taste, we hypothesized that Sjögren’s syndrome patients with less severe dry-mouth experience will prefer other flavours than patients with more severe dryness. In addition, we hypothesized that Sjögren’s patients will have more objections against the presence of specific animal-based ingredients than non-animal–based products.

## Material and methods

A cross-sectional study was performed among Sjögren’s syndrome patients who visited the website of the Dutch Sjögren Patients Federation (Dutch: Nederlandse Vereniging van Sjögren Patiënten). Volunteers could anonymously fill in the questionnaire described below during a period of 7 weeks. Only volunteers with diagnosed Sjogren’s syndrome were eligible to fill in the questionnaire.

The local Ethics Review Committee of the Academic Centre for Dentistry Amsterdam (ACTA) confirmed that the Medical Research Involving Human Subjects Act (WMO) did not apply to this study (protocol number 2017001). The reporting of this study conforms to the Strengthening the Reporting of OBservational studies in Epidemiology (STROBE) statement [15].

A priori sample size calculation was performed using G*Power software, version 3.1.9.4 (Heinrich-Heine-Universität Düsseldorf, Düsseldorf, Germany); with a medium effect size (0.5) and a power of 80%, 148 participants were needed.

### Study variables

The questionnaire, developed for this study, consisted of eight parts. First, several general questions with regard to age, sex, and year when Sjögren’s syndrome had been diagnosed by a physician.

The second part was the internationally accepted and validated Xerostomia Inventory (XI), consisting of 11 items on a 5-point Likert scale ranging from 1 = “Never” to 5 = “Very often”. The items concern patients’ oral dryness and mouthfeel. Per item, patients indicate how often they experience problems regarding mouthfeel and oral dryness. The scores of the 11 items are summed to produce a total XI score that ranges between 11 (no xerostomia) and 55 (extreme xerostomia) [16]. XI had showed adequate content and concurrent validity [16].

The remaining parts of the questionnaire contained questions regarding various product characteristics of hypothetical new saliva substitutes.

The third part explored the importance of different functions of saliva substitutes. The patients could indicate the importance of each function by using a 5-point Likert scale, ranging from 1 = “Unimportant” to 5 = “Very important”. All investigated possible functions of salivary substitutes are presented in Table 1.

**Table 1:** Sjögren’s syndrome patients’ opinon regarding the importance of the potential functions of saliva substitutes, using a Likert scale (from 1 = “Unimportant” to 5 = “Very important”). Data are expressed as as mean score with standard deviation (*SD*) and median scores with the corresponding interquartile range (*IQR*). *N* indicates the number of participants in each group

Possible functions	Total mean ± SD (*N*)	Total median ± IQR
Helps to prevent tooth decay	4.03 ± 1.36 (*N* = 59)	5.0 ± 3.0–5.0
Helps painful swallowing	4.15 ± 1.20 (*N* = 59)	5.0 ± 4.0–5.0
Provides fast alleviation of dry mouth	4.49 ± 1.01 (*N* = 59)	5.0 ± 4.0–5.0
Gives prolonged alleviation of dry mouth	4.64 ± 0.74 (*N* = 59)	5.0 ± 4.0–5.0
Protects the mucosa	4.49 ± 0.88 (*N* = 59)	5.0 ± 4.0–5.0
Treats bleeding gingiva	3.54 ± 1.29 (*N* = 59)	4.0 ± 3.0–5.0
Facilitates speaking	4.48 ± 1.02 (*N* = 59)	5.0 ± 4.0–5.0
Improves the taste	3.95 ± 1.15 (*N* = 58)	4.0 ± 3.0–5.0
Available in different flavours	3.09 ± 1.38 (*N* = 59)	3.0 ± 2.0–4.0
Stimulates saliva secretion	4.41 ± 0.97 (*N* = 59)	5.0 ± 4.0–5.0
Neutralizes harmful bacteria	4.63 ± 0.79 (*N* = 59)	5.0 ± 4.0–5.0
Optimizes the mouth flora	4.60 ± 0.77 (*N* = 58)	5.0 ± 4.0–5.0
Contains natural saliva enzymes	4.17 ± 1.09 (*N* = 58)	5.0 ± 3.8–5.0
Gives a balanced pH	4.29 ± 0.86 (*N* = 58)	4.5 ± 4.0–5.0
Is practical and handy in use	4.46 ± 0.95 (*N* = 57)	5.0 ± 4.0–5.0
Can be used with little effort	4.40 ± 0.90 (*N* = 58)	5.0 ± 4.0–5.0
Can be used unnoticed	2.98 ± 1.54 (*N* = 59)	3.0 ± 2.0–4.0

The fourth part consisted of a question about the preferred consistency of saliva substitutes; thin-watery or thick-liquid-like or gel-like.

The fifth part explored how much the patients object the presence of certain ingredients in saliva substitutes using 5-point Likert scales, ranging from 1 = “No objection” to 5 = “Insurmountable objections”. Table 2 presents all potential ingredients investigated.

**Table 2:** Sjögren’s syndrome patients’ objections against certain ingredients in saliva substitutes, using a 5-point Likert scale (from 1 = “No objection” to 5 = “Insurmountable objections”). Data are expressed as mean score with standard deviation (*SD*) and as median scores with the corresponding interquartile range (*IQR*). *N* indicates the number of participants in each group

Potential ingredients	Total mean ± SD (*N*)	Total median ± IQR
Alcohol	3.39 ± 1.46 (*N* = 57)	3.0 ± 2.0–5.0
Preservatives	3.14 ± 1.38 (*N* = 57)	3.0 ± 2.0–4.5
Fluoride	3.03 ± 1.47 (*N* = 57)	1.0 ± 1.0–3.0
Urea	2.82 ± 1.17 (*N* = 57)	3.0 ± 2.0–3.0
Foaming agents	3.30 ± 1.35 (*N* = 57)	3.0 ± 2.5–4.0
Artificial sweeteners	3.40 ± 1.52 (*N* = 57)	4.0 ± 2.0–5.0
Gluten	2.67 ± 1.57 (*N* = 57)	3.0 ± 1.0–4.0
Natural enzymes	1.72 ± 1.22 (*N* = 57)	1.0 ± 1.0–2.5
Vegetable-based ingredients	1.58 ± 1.12 (*N* = 57)	1.0 ± 1.0–2.0
Ingredients from chicken eggs	2.32 ± 1.38 (*N* = 57)	2.0 ± 1.0–3.0
Ingredients from cattle	2.58 ± 1.40 (*N* = 57)	3.0 ± 1.0–3.5
Ingredients from pigs	3.04 ± 1.49 (*N* = 57)	3.0 ± 1.5–4.5
Ingredients from fish	2.82 ± 1.42 (*N* = 56)	3.0 ± 1.0–4.0

The sixth part consisted of an item regarding the desired flavour of saliva substitutes. A 5-point Likert scale was used to indicate the importance of the availability of each flavour, ranging from 1 = “Unimportant” to 5 = “Very important”. The desired flavours investigated are presented in Table 3.

**Table 3:** Sjögren’s syndrome patients’ opinion regarding the desired flavours of saliva substitutes, using a Likert scale (from 1 = “Unimportant” to 5 = “Very important”). Patients were stratified in subgroups based on their age and severity of xerostomia. For the total study population, the data are expressed as mean score with standard deviation (*SD*) and as median scores with the corresponding interquartile range (*IQR*). For both age groups and XI-groups, the mean scores with SD were reported. *N* indicates the number of participants in each group

Possible flavours	Total mean ± SD (*N*)	Total median ± IQR	Birth year ≤ 1958 (*N*)	Birth year ≥ 1959 (*N*)	*p*-value*	XI-score ≤ 46 (*N*)	XI-score ≥ 47 (*N*)	*p*-value*
Strawberry	1.81 ± 1.27 (*N* = 57)	1.0 ± 1.0–3.0	1.77 ± 1.24 (*N* = 26)	1.84 ± 1.32 (*N* = 31)	0.55	1.79 ± 1.18 (*N* = 29)	1.82 ± 1.39 (*N* = 28)	0.54
Apple	2.03 ± 1.36 (*N* = 58)	1.0 ± 1.0–3.0	1.89 ± 1.31 (*N* = 27)	2.16 ± 1.42 (*N* = 31)	0.29	2.17 ± 1.47 (*N* = 29)	1.90 ± 1.26 (*N* = 29)	0.40
Banana	1.82 ± 1.31 (*N* = 57)	1.0 ± 1.0–3.0	1.77 ± 1.24 (*N* = 26)	1.87 ± 1.38 (*N* = 31)	0.49	1.86 ± 1.27 (*N* = 29)	1.79 ± 1.37 (*N* = 28)	0.38
Blueberry	2.09 ± 1.43 (*N* = 56)	1.0 ± 1.0–3.0	2.08 ± 1.50 (*N* = 26)	2.10 ± 1.40 (*N* = 30)	0.64	2.41 ± 1.57 (*N* = 29)	1.74 ± 1.20 (*N* = 27)	0.03
Lemon	2.31 ± 1.50 (*N* = 58)	1.5 ± 1.0–4.0	2.33 ± 1.59 (*N* = 27)	2.29 ± 1.44 (*N* = 31)	0.83	2.69 ± 1.54 (*N* = 29)	1.93 ± 1.39 (*N* = 29)	0.05
Cola	1.49 ± 1.12 (*N* = 57)	1.0 ± 1.0–1.0	1.54 ± 1.21 (*N* = 26)	1.45 ± 1.06 (*N* = 31)	0.56	1.59 ± 1.24 (*N* = 29)	1.39 ± 0.99 (*N* = 28)	0.34
Liquorice	1.81 ± 1.37 (*N* = 57)	1.0 ± 1.0–2.5	1.81 ± 1.36 (*N* = 26)	1.81 ± 1.40 (*N* = 31)	0.75	1.79 ± 1.37 (*N* = 29)	1.82 ± 1.39 (*N* = 28)	0.66
Menthol/spearmint	3.57 ± 1.55 (*N* = 58)	4.0 ± 3.0–5.0	2.96 ± 1.53 (*N* = 27)	4.10 ± 1.38 (*N* = 31)	0.003	3.66 ± 1.50 (*N* = 29)	3.48 ± 1.62 (*N* = 29)	0.58
No flavour	3.91 ± 1.58 (*N* = 57)	5.0 ± 3.0–5.0	3.88 ± 1.68 (*N* = 26)	3.94 ± 1.53 (*N* = 31)	0.65	3.79 ± 1.66 (*N* = 29)	4.04 ± 1.53 (*N* = 28)	0.89
Neutral flavour	3.98 ± 1.47 (*N* = 58)	5.0 ± 3.0–5.0	3.93 ± 1.57 (*N* = 27)	4.03 ± 1.40 (*N* = 31)	0.74	3.52 ± 1.64 (*N* = 29)	4.45 ± 1.12 (*N* = 29)	0.02

^*^p-value of the Mann–Whitney *U* test

The seventh part of questionnaire was about potential side effects of saliva substitutes. For each side effect, the patient could indicate if they would experience it as unpleasant by using a 5-point Likert scale ranging from 1 = “Not unpleasant” to 5 = "Very unpleasant”. Table 4 presents the investigated potential side effects of salivary substitutes.

**Table 4:** Sjögren’s syndrome patients’ opinion regarding potential side effects of saliva substitutes, using a 5-point Likert scale (1 = “Not unpleasant” to 5 = “Very unpleasant”). Data are expressed as mean score with standard deviation (*SD*) and as median scores with the corresponding interquartile range (*IQR*). *N* indicates the number of participants in each group

Potential negative effects and side effects	Total mean ± SD (*N*)	Total median ± IQR
Causing discoloration of the teeth	4.59 ± 1.03 (*N* = 58)	5.0 ± 5.0–5.0
Causing discoloration of the oral mucosa	4.14 ± 1.12 (*N* = 58)	5.0 ± 3.0–5.0
Having a bitter taste	4.47 ± 0.90 (*N* = 58)	5.0 ± 4.0–5.0
Having an aftertaste	4.22 ± 0.94 (*N* = 58)	4.5 ± 4.0–5.0
Using the product multiple times a day	2.45 ± 1.33 (*N* = 58)	3.0 ± 1.0–3.0

Finally, a question was included about the preferred method of administration of the saliva substitutes, whereby patient could choose a mouth gel, a mouth spray, an oral rinse, or a tablet.

### Data analysis

The data were statistically analysed with SPSS, version 28.0 (IBM Corp SPSS statistics, Armonk, NY, USA). The Shapiro–Wilk test was used to assess the normality of the data. As not all variables were normally distributed, the data are presented as medians and their interquartile range (IQR). To clarify relatively small differences, the mean and standard deviation (SD) are also reported.

The respondents were dichotomized based on their age and the severity of their xerostomia. The two xerostomia groups were used to test the hypothesis whether Sjögren’s syndrome patients with less severe dry-mouth experience prefer other flavours than patients with more severe dryness. The median of these two parameters was used to divide them into two groups: birth year ≤ 1958 versus birth year ≥ 1959 and mouth dryness with a XI-score ≤ 46 versus mouth dryness with a XI-score ≥ 47. A Mann–Whitney *U* test was used to explore whether the subgroups of respondents varied based on their respective answers.

All significance levels (*α*) were set at 0.05.

## Results

At the time the questionnaire was distributed to the patients, the patients’ association had 2115 members. In the period when the questionnaire was available online, the association’s website was visited by 1485 people. During this period, 59 Sjögren’s syndrome patients completed the questionnaire. Almost all respondents were women (*N* = 58, 98%). The mean age of the respondents was 55.7 ± 12.0 years, ranging from 25 to 79 years. The respondents reported that the Sjögren’s syndrome had been diagnosed between 1 and 36 years ago. The total XI-score of all patients had a median of 47.0 with IQR of 43.0–51.0.

Table 1 describes the opinion of Sjögren’s syndrome patients regarding the importance of different functions of saliva substitutes. Most of the possible functions were considered important (score ≥ 4), while functions such as “provides fast alleviation of dry mouth”, “gives prolonged alleviation of dry mouth”, “protects the mucosa”, “facilitates speaking”, “neutralizes harmful bacteria”, and “optimizes the mouth flora” were considered very important (score ≥ 4.5). On the other hand, functions such as “available in different flavours” and “can be used unnoticed” were considered relatively less important. The two age groups did not show any significant differences regarding possible functions of saliva substitutes (Mann–Whitney *U* test *p* > 0.05). Respondents with a more severe xerostomia, indicated by a higher XI-score, considered the function “gives a prolonged alleviation of dry mouth” more important than the lower XI-group (mean = 4.73 ± 0.83, median = 5.0 ± 5.0–5.0, *N* = 30, versus mean = 4.55 ± 0.63, median = 5.0 ± 4.0–5.0, *N* = 29, Mann–Whitney *U* test *p* < 0.05). All other functions did not show any significant difference for the two XI-groups.

As for the consistency of salivary substitutes, most of the respondents preferred thin-watery consistency (52.5%) followed by gel-like consistency (33.9%). Only 8.5% of respondents preferred a thick-liquid consistency. Age or XI-groups did not influence the preference of the consistency.

In Table 2, the objections of the respondents against the presence of certain ingredients are reported. The respondents mainly objected against the presence of “artificial sweeteners”, “alcohol”, “foaming agents”, and “preservatives”. They objected less against the presence of “vegetable-based ingredients”, “natural enzymes”, and “fluoride”. The two XI-groups only showed a significant difference with regard to the objections against vegetable-based ingredients (Mann–Whitney *U* test *p* < 0.05). Respondents with relatively low xerostomia (mean = 1.31 ± 0.89, median = 1.0 ± 1.0–1.0, N = 29) had less objections against the presence of vegetable based ingredients than the respondent with more severe xerostomia (mean = 1.86 ± 1.27, median = 1.0 ± 1.0–3.0, *N* = 28).

The opinion of the respondents regarding the desired flavours of salivary substitutes is reported in Table 3. Highly preferred flavours were a “neutral flavour”, “no flavour”, and “menthol/spearmint flavour”, whereas the flavours “cola”, “liquorice”, and “strawberry” were the least popular. There was a significant difference between the two age groups with regard to preferences of flavours (Mann–Whitney *U* test *p* < 0.05); the younger respondents preferred “menthol/spearmint” flavour more than the older age group. The two groups with different levels of xerostomia also showed significant differences (Mann–Whitney *U* test *p* < 0.05). The respondents with relatively low xerostomia (XI-score ≤ 46) preferred the use of flavour “blueberry” more than the respondents with more severe xerostomia (XI-score ≥ 47). On the other hand, respondents in the XI ≥ 47 group preferred the use of “neutral flavours” in salivary substitutes significantly more than respondents with a relatively low xerostomia.

Table 4 depicts the opinion of respondents regarding potential side effects of the use of saliva substitutes. Major objections were against saliva substitutes “causing discoloration of the teeth” and ones “having a bitter taste”. The least objections were about using saliva substitutes multiple times a day. The two age groups only differed significantly with regard to “causing discoloration of the teeth” (Mann–Whitney *U* test *p* < 0.05), whereby the younger age group (mean = 4.74 ± 0.89, median = 5.0 ± 5.0–5.0, *N* = 31) had more objections than the older age group (mean = 4.25 ± 1.40, median = 5.0 ± 4.0–5.0, *N* = 28).

Finally, Table 5 presents the preferred method of administration. The respondents preferred a mouth spray followed by a mouth gel or an oral rinse. A minority of the respondents preferred a tablet. These preferences did not differ significantly for the two age groups and the two XI-groups (Mann–Whitney *U* test *p* > 0.05).

**Table 5:** Sjögren’s syndrome patients’ opinion regarding the preferred method of administration. Data are presented as percentages

Method of administration	Percentage
Mouth spray	45.5
Mouth gel	23.6
Oral rinse	23.6
Tablet	7.3

## Discussion

The present study was designed to explore criteria for new saliva substitutes according to the preferences of Sjögren’s syndrome patients. The most ideal saliva substitute has thin-watery consistency in spray form, with a neutral flavour and providing a prolonged alleviation of dry mouth. Besides, it preferably should not contain artificial sweeteners or alcohol, and should not have a bitter taste and not cause discoloration of the teeth.

Most of the respondents of the present study were female (98%) with average age of 55.7 ± 12.0 years and with severe dry-mouth complaints, as indicated by the high average XI-score (47.0 ± 43.0–51.0). This overrepresentation is in line with the female to male ratio of Sjögren’s syndrome, which varies between 20:1 and 9:1 [17]. The average age and the severity of oral dryness in the current study are also comparable with other studies that included Sjögren’s syndrome patients with dry-mouth complaints [7, 18]. The average age in these previous studies varied between 61.7 ± 14.0 and 64 ± 10 years. As for the severity of xerostomia, the mean XI-scores in these previous studies were between 44.0 ± 37.0–49.8 and 45.0 ± 38.0–48.5 [7, 18]. In summary, this suggests that the respondents in the current study form a good representation of Sjögren’s syndrome patients in the Dutch population.

Several systematic reviews have reported that the effectiveness of currently available saliva substitutes for the relief of dry mouth seems to be limited [4, 6, 19]. For this reason in the present study, the Sjögren’s syndrome patients indicated that prolonged alleviation of dry mouth is the most essential function of saliva substitutes. Unfortunately, most of the available saliva substitutes now provide only a temporary relief [9]; as the lubrication time of a typical saliva substitute, such as Dentaid Xeros, is around 0.5 min [9]. However, recently a promising new supercharged polypeptide-based salivary lubrication enhancer has been reported which could prolong the lubrication time up to 21 ± 7.3 min [9].

When developing a new saliva substitute, it is also important to try to mimic the complex biological properties of natural saliva, including neutralizing harmful bacteria and optimizing the mouth flora. Many Sjögren’s syndrome patients with a reduced salivary flow have alterations in the composition of the oral bacterial plaque despite good oral hygiene measures [20, 21], which causes an increased risk of caries and candidiasis [20, 21]. Moreover, the salivary pH, bicarbonate concentration, and buffer capacity were significantly lower in the Sjögren’s syndrome patients compared to healthy controls [21]. Besides an increased high caries risk, these patients have also a higher risk of tooth demineralisation, as they experience a greater decline in salivary pH after exposure to acidic challenges. These factors might explain the urge of Sjögren’s syndrome patients for a salivary substitute that “neutralizes harmful bacteria” and “optimizes the mouth flora”.

Saliva plays a major role in taste perception, as the hypotonicity of unstimulated saliva allows the taste buds to perceive different tastes without being masked by normal plasma sodium levels [22]. Moreover, saliva is very important for the solubilization of flavours in saliva, for the chemical interaction between flavours and salivary ingredients, and for the dilution and/or the diffusion of flavours in saliva [23]. Based on these factors, it is conceivable that taste sensitivity is easily affected by changes in saliva [23], especially in Sjögren’s syndrome patients with a reduced unstimulated salivary flow rate [24–30] and altered rheological properties of saliva [31]. This altered taste sensitivity may explain why these patients had objections against the presence of “artificial sweeteners” and “alcohol” and why they preferred a “neutral flavour” or “no flavour” at all. Previous studies showed that an unpleasant taste is a major reason for Sjögren’s syndrome patients to discontinue the use of saliva substitutes [12]. Sjögren’s syndrome patients having sicca syndrome are recommended to avoid alcohol [32], which may explain why patients prefer saliva substitutes without alcohol. Given these reasons, it is important to develop new saliva substitutes with a “neutral flavour” without “artificial sweeteners” nor “alcohol”. In contrast to our expectation described in the “Introduction”, the presence of specific animal-based ingredients seems of very limited importance, compared to other ingredients such as “artificial sweeteners” or “alcohol”.

Sjögren’s syndrome patients reported major objections against discoloration of the teeth or the oral mucosa as potential side effects of the use of saliva substitutes. Discoloration was not mentioned in a study reporting side effects of some saliva substitutes [33]. Possibly, these objections against discoloration might be related that white teeth are important for people in general, as demonstrated by others [34].

In the current study, the flavours of the available saliva substitutes such as “apple”, “lemon”, and “strawberry” were the least preferred, although a previous study has showed that a malic acid (“apple acid”) containing spray significantly stimulated salivary flow rate in patients using antihypertensive medication and improved their xerostomia [35]. However, this positive effect on oral dryness will be less or completely absent in Sjögren’s syndrome patients with an advanced disease process.

As mentioned in the “Introduction”, the severity of the dry-mouth feeling, as measured with XI, may influence the preference of desired flavours. Patients with low xerostomia preferred the use of the flavour “blueberry” more than the respondents with more severe xerostomia. On the other hand, respondents’ severe xerostomia preferred the use of “neutral flavours” in salivary substitutes more. This confirms the hypothesis that severity of oral dryness may play a major role in the preferred saliva substitute flavours.

A possible limitation of the current study is that the reported preferences for saliva substitutes are only based on the opinion of Sjögren’s syndrome patients. However, saliva substitutes are also used by patients suffering from oral dryness due to other conditions, including patients using xerogenic medications or polypharmacy, and patients irradiated in the head and neck region [2, 36–38]. Further research should investigate whether the preferences of these other dry-mouth patients are similar to those of Sjögren’s syndrome patients.

Another possible limitation of the current study is that the Sjögren’s syndrome patients, who filled in this questionnaire, may be more interested in oral health than other Sjögren’s syndrome patients, or suffer from more severe xerostomia. This may have resulted in an above-average oral dryness which may have affected the preferences of new saliva substitutes. Besides, it is unknown which diagnosis criteria have been used by the patients’ physician to establish the diagnosis of Sjögren’s syndrome, and whether they suffered from primary or secondary Sjögren’s disease.

Finally, another limitation is that the actual number of participants in the current study is lower than the number calculated a priori. This indicates that the power of the current study is relatively low, and so all results in which no significant differences were found between the two age or XI-groups should be interpreted with caution.

## Main conclusion

The current study has identified preferences criteria of Sjögren’s syndrome patients regarding various product characteristics for new saliva substitutes. The most ideal saliva, according to Sjögren’s syndrome patients, has thin-watery consistency in spray form with a neutral flavour and providing long alleviation of dry mouth complaints. Besides, it should not contain artificial sweeteners or alcohol, and should not have a bitter taste or cause discoloration of the teeth.
